# Multi-Parametric Clustering for Sensor Node Coordination in Cognitive Wireless Sensor Networks

**DOI:** 10.1371/journal.pone.0053434

**Published:** 2013-02-13

**Authors:** Xiao Yu Wang, Alexander Wong

**Affiliations:** Department of Systems Design Engineering, University of Waterloo, Waterloo, Canada; Umeå University, Sweden

## Abstract

The deployment of wireless sensor networks for healthcare applications have been motivated and driven by the increasing demand for real-time monitoring of patients in hospital and large disaster response environments. A major challenge in developing such sensor networks is the need for coordinating a large number of randomly deployed sensor nodes. In this study, we propose a multi-parametric clustering scheme designed to aid in the coordination of sensor nodes within cognitive wireless sensor networks. In the proposed scheme, sensor nodes are clustered together based on similar network behaviour across multiple network parameters, such as channel availability, interference characteristics, and topological characteristics, followed by mechanisms for forming, joining and switching clusters. Extensive performance evaluation is conducted to study the impact on important factors such as clustering overhead, cluster joining estimation error, interference probability, as well as probability of reclustering. Results show that the proposed clustering scheme can be an excellent candidate for use in large scale cognitive wireless sensor network deployments with high dynamics.

## Introduction

The use of medical sensor systems such as electroencephalography (EEG), electrocardiography (ECG), blood pressure monitors, and glucose monitors for monitor the vital signs of patients have long been a staple of the modern healthcare establishment. However, such medical sensing systems have been largely wired and tethered to a specific location, which results in not only a mess of wires that can affect patient comfort levels, but also requires a wired infrastructure that is inconvenient for clinical personnel as well as restricts patient mobility [1]. This restriction in mobility would be considered particularly disadvantageous in large disaster response situations [2], [3]. With the advent of advanced wireless communication technologies and portable sensing devices, there is now an increasing demand for wireless sensor networks (WSNs) to address some of these limitations. For example, patients within hospitals would be able to move from location to location without having to be reconnected, thus allowing for uninterrupted real-time patient monitoring. In the case of large disaster response scenarios, a large number of victims who are scattered a random locations can be monitored simultaneously at the disaster location. As such, there are a large number of healthcare scenarios where wireless sensor networks can enable a much more robust and versatile health monitoring environment than conventional wired systems. Despite the great number of advantages of such wireless sensor networks for healthcare monitoring, there are still a number of technical challenges that must be resolved, and as such new approaches for the deployment and management of such networks are required.

A key challenge associated with the deployment of wireless sensor networks for healthcare monitoring purposes is the coordination and management of a large number of randomly deployed sensor nodes. Proper coordination and management is necessary for reducing the communication bandwidth and energy requirements, as well as interference of a large number of sensor nodes working simultaneously within a common wireless sensing network. One effective approach to tackling this issue is to group sensor nodes into clusters to facilitate for improved coordination as well as reduced resource usage. For example, it is more efficient resource-wise for designated sensor nodes (“clusterheads”) to collect sensing data from neighboring sensor nodes within clusters and then transmit the data back to a central source, than for individual sensor nodes to transmit to a central source in a direct manner. Furthermore, in the case of cognitive wireless sensor networks (CWSNs) [4], [5], clustering also aids in the coordination of cognitive sensor nodes so that the unlicensed spectrum resources can be better utilized for improved coverage and quality of service, while at the same time reducing interference. This is particularly important in a large disaster scenario, where there are a large number of sensing nodes being deployed (as well as other mobile communication devices) that can starve the primary spectrum resources and result in communications failure.

There has been extensive research on clustering techniques in both conventional WSNs and Mobile Ad Hoc Networks (MANETs). In conventional WSNs, clustering is utilized primarily to reduce energy consumption during the data gathering process, which is critical for extending the lifetime of such networks since sensor nodes often have limited available energy [6], [7]. As such, the criteria for clustering in WSNs primarily depends on energy constraints. In MANETs, the main purpose for clustering is to maintain a certain level of system performance in the context of large numbers of high mobility wireless nodes. Given this type of scenario, clustering for MANETs relies more on the overall topology of the network formed by mobile nodes to determine the cluster formation [8], [9].

In this work, we explore the use of clustering to aid in the coordination of cognitive sensor nodes in cognitive wireless sensor networks. The spectrum resource that we utilize in this work is multiple spatially and temporarily available subbands, which is different from the statically assigned single radio channel of conventional WSNs and MANETs. The dynamic nature of spatially and temporarily available subbands introduces challenges of returning legitimate users on the already identified available subbands, and the characteristics of the returning legitimate users may be very different on each subbands and result in interference. Therefore, existing techniques may not work well for such cognitive wireless sensor networks and hence a new approach to designing clustering for cognitive sensor nodes within such a network is required.

The main contributions of this paper are summarized as follows.

1. A novel approach for dynamically clustering cognitive sensor nodes based on weighted Fuzzy C-means (FCM) [23] that takes into consideration multiple important network parameters such as channel availability, interference characteristics, and topological characteristics is introduced to allow for more reliable and efficient communication.
2. A probabilistic approach to defining cluster membership is employed instead of the conventional deterministic approach of fixed cluster memberships. This approach allows cognitive sensor nodes to join and switch between different clusters for better connection, thus allowing better spectrum efficiency to be achieved.
3. A set of cluster formation, cluster joining, cluster switching, and resolution mechanisms is introduced.

Simulation results shows great potential for achieving improved performance when compared with existing clustering algorithms in terms of clustering overhead, cluster joining estimation error, interference probability, and probability of reclustering.

The rest of this paper is organized as follows. First, the related work is reviewed. The system model is then presented, and the proposed clustering scheme is described. Performance evaluation results are then presented and conclusions are drawn.

### Related Work

Given the relative infancy of the research area, there are only a few related works on clustering for multichannel ad-hoc networks such as cognitive wireless sensor networks. In general, channel availability is the most popular network parameter for clustering nodes. Most of these schemes [10]–[13] assume or imply that spectrum availability does not change during the cluster formation procedure. In [10], clusters are formed based on a single available channel, where nodes compete to be the clusterhead after detecting no messages on the channel. The successful clusterhead then issues a beacon to control the channel access of the cluster. In [12], [13], a recursive distributed voting scheme is used to dynamically select a common channel, which is labeled with the highest connectivity with a neighborhood. Any new joining nodes eavesdrops the control messages to determine which cluster to join. Since this approach attempts to cluster users through a single common channel, changes in primary user activities on this channel can result in the disconnection of users in the group, which leads to frequent reclustering to ensure full neighborhood connectivity [14].

To reduce the amount of reclustering, Lazos *et al*. formulated the clustering problem as a maximum edge biclique problem, which is solved by a spectrum-opportunity based clustering algorithm [14]. In this approach, the nodes are clustered based on similar channel availability, which allows the nodes to choose a control channel from a group of available channels. Furthermore, the nodes can migrate to another control channel without the need for reclustering if the current control channel becomes occupied due to primary user activities. However, since only channel availability is taken into consideration when determining the clustering of nodes, this approach can potentially be sensitive to the presence of interference between different nodes.

### System Model

In this study, the CWSNs consist of cognitive sensor nodes that collect real-time data over a large area and needs that data to be transmitted reliably in a continuous manner, such as for healthcare applications such as real-time monitoring of patients in hospital and large disaster response environments. While such nodes may operate regularly in the unlicensed industrial, scientific and medical (ISM) bands, as most WSNs do, the ISM band is also used by an increasing number of wireless devices operating on technologies such as Bluetooth, Wi-Fi, and near field communication (NFC). As such, not only is the unlicensed ISM band spectrum resources become increasing scarce, co-existence issues arise between different devices using different protocols and technologies all trying to operate within the unlicensed ISM band. Therefore, in this study, we consider the cognitive sensor nodes being deployed in the CWSNs to be equipped with extra capability for spectrum sensing and opportunistic accessing to already-licensed spectrum bands for other services, such as any television (TV) bands within 400–600 MHz and Ultra High Frequency (UHF) TV bands, which has recently been made available by government regulators such as the Federal Communications Commission (FCC). Therefore, in the situation where the unlicensed ISM spectrum has been consumed by on-going data traffic or results in interference, the cognitive sensor nodes can make use of the additional white space.

In this study, there are a total of 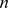 primary users, and 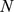 cognitive sensor nodes. The cognitive sensor nodes are not necessary within the radio transmission range of each other. The primary users are designated as the legitimate users and are statically allocated in the TV band. In this same spectrum, each of the cognitive sensor nodes has the additional capability to sense and opportunistically access spatiotemporally available spectrum resources in a secondary manner.

### 3.1 Channel Model

The spectrum available for opportunistic access is divided into 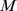 non-overlapping subbands, denoted as 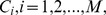 with 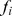 denoting the center frequency of 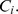 A channel is a single subband and the unit of spectrum usage. The radio propagation of these channels is assumed to be subject to small scale Rayleigh fading, which has been commonly taken to describe the rapid fluctuation of radio signal over a short period of time or transmission distance [15]. Mathematically, given a transmitter-receiver pair and the transmitter power 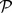, the received signal strength is given as:
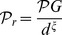
(1)where 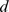 is the distance between the transmitter and the receiver, 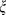 is the path loss exponent, and 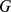 is a comprehensive channel factor including a random value of a chi-square distribution with two degrees of freedom modeling the Rayleigh fading, as well as antenna gain factor. Where the primary users are randomly located around a reference cognitive sensor node, the total received power at the reference cognitive sensor node on channel 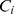 from primary transmissions is given as [16]:
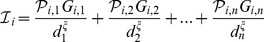
(2)where 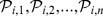 denotes the power of ongoing transmission by primary users indexed as 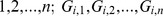 denotes the channel factor of corresponding primary users; and 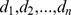 denotes the distance between the cognitive sensor node to the primary users.

### 3.2 Spectrum Sensing Model

In the Rayleigh fading channel model, each cognitive sensor node detects the presence of primary users independently of other cognitive sensor nodes. Since it is very hard to correctly distinguish a faded signal of a primary user from the environmental noise and hence the existence of primary users in the presence of channel fading, a number of feature detection techniques have been proposed to differentiate the noise energy from the signal energy, such as cyclostationary feature detection [17] and pilot signal detection. However, comparing to the widely used energy detection, the main disadvantages of feature detection method are long observation time, computational complexity, and greater energy consumption. Moreover, some primary user signals exhibit no stable and common features, such as wireless microphone signals operating in TV white space spectrum [18]. Therefore, in this work, energy detection is conservatively assumed to obtain the presence of the primary users in a fast but relatively inaccurate manner. Given a certain probability of false alarm, 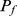, the average detection probability 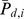 of channel 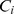 can be approximated [19].

### 3.3 Interference Model

Branching off interference modeling work in cognitive networks [20], [21], in the interference model, the victim receiver is defined as a primary user which is subject to interference by cognitive sensor nodes. Three of the main causes of such interference are considered. The first cause of interference being considered is that primary users are spatially distributed within the interference range 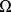 of the cognitive sensor nodes, following a probability distribution function 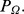 For example, in the simple case where the primary users are uniformly distributed within 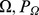, can be defined as the ratio between the area of 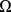 and the area of primary user network coverage.

The second cause of interference being considered is that that primary users may return to channels that were previously identified as available but is currently being occupied by cognitive sensor nodes. It is assumed that the spectrum usage of primary users on channel 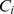 follows an M/G/1 model [22], where the primary users arrive on channel 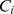 according to a Poisson process with rate 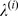, and the busy period of usage is on an arbitrary distribution. Therefore, the probability of the earliest returning primary user during the transmission period of cognitive sensor node can be estimated as [22].
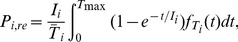
(3)where 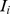 is the average idle period in the primary user network for 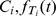 is the probability density function of cognitive sensor node transmission time for 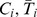 is the average transmission time of 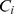, and 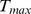 is the maximum allowable transmission time.

The third cause of interference being considered is that, technically, due to the detection sensitivity, the cognitive sensor nodes are not able to identify severely faded primary user signals, and thus leading to undesirable access to unavailable channels that are being used by primary users. The corresponding probability can be determined by 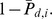


Based on these three causes, interference to the primary users by the cognitive sensor nodes can be a result of two scenarios: 1) detection error in the interference range of the cognitive sensor nodes, 2) returning primary users to the successfully identified channel without any false alarm, i.e., 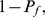 within the interference range of the cognitive sensor node; therefore, the probability of interference can be evaluated as
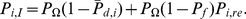
(4)


### 3.4 Discovery Model

Note that before cluster formation, cognitive sensor nodes do not directly communicate with each other. However, the cognitive sensor nodes can communicate with macrocell base stations wirelessly (though briefly to limit communication overhead). This facilitates the discovery of the other cognitive sensor nodes. Through brief information exchanges with macrocells, the cognitive sensor nodes are aware of information about other cognitive sensor nodes. In particular, the information about a cognitive sensor node 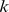 includes its location 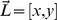 denoted the spatial coordinates, available channels 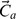 with index 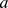, as well as the corresponding interference characteristics 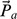 of available channels with index 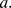 This information can be represented by the vector 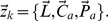 The overall information of all cognitive sensor nodes is denoted as 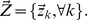 The information of available channels and interference characteristics is time sensitive and is the key to the proper coordination among cognitive sensor nodes. We will take them into consideration while designing the clustering scheme.

## Methods

### 4.1 Overview

**Figure 1:**
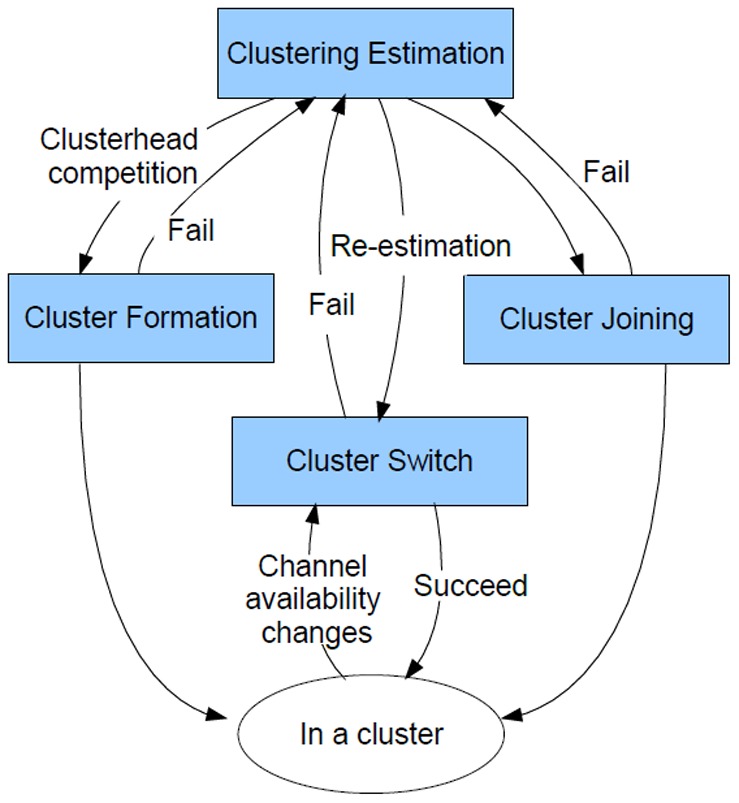
Stages of proposed clustering scheme.

The proposed multi-parametric clustering scheme can be divided into four main stages: i) *clustering estimation*, ii) *cluster formation*, iii) *cluster joining*, and iv) *cluster switching*. The interaction of each stage of the proposed scheme is shown in Fig. 1. In the clustering estimation stage, each cognitive sensor node autonomously identifies the possible clusters within the network as well as its membership via weighted Fuzzy C-means clustering (FCM) [23]. Based on the clustering estimation, the selected candidate cognitive sensor nodes enter into the cluster formation stage, and compete for clusterhead that provide service to the individual clusters, as well as identify the common channels upon which the service can be provided. The remaining cognitive sensor nodes enter into the cluster joining stage, where the joining cognitive sensor nodes decide upon which cluster to join and the corresponding mechanism to employ. Finally, in the cluster switching stage, cognitive sensor nodes switch fast to backup clusters based on the cluster membership estimated in the clustering estimation stage to avoid returning primary users.

The following subsections describe the detailed mechanisms of clustering estimation, cluster formation, cluster joining, as well as resolution of returning primary users.

### 4.2 Clustering estimation with weighted FCM

Clustering with low communication overhead is challenging, especially under the situation where the spectrum resource is scarce and network connectivity are rapidly changing [24]. To make this demand, the proposed scheme employs and modifies Fuzzy C-means (FCM) [23] clustering, which is relatively fast and, not only does it allow for better handling and modeling of imprecise data with large variability by allowing for cluster mixtures in a probabilistic manner, which is important given the set of network parameters being considered, but also can be implemented distributively on individual cognitive sensor nodes to reduce the communication overhead. The information 
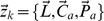
 pertaining to the cognitive sensor node 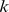 contains location information 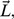 as well as time sensitive information about multiple network parameters, such as channel availability 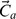 and the corresponding interference characteristics 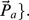 Therefore, reliability factors are required to weight the obtained information. In terms of channel availability, it is easy to assume that the use of channels by the primary users does not change during certain period of time, such as a time slot or a cycle of updating period. However, it is often not practical and the reliability of usage information fades as time elapses. To avoid a binary response (e.g. reliable or not), a soft thresholding strategy is employed on a set of available channels 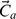 to ensure tighter estimates based on Poisson process of primary user arrival 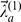 by using
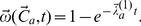
(5)


Moreover, in terms of interference characteristics, the time sensitivity has been reflected in the estimation of the primary user arrival in Eq. (3). The value of the arrival rate 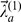 on the set of available channels 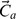 can be estimated based on a sliding observation window 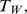 where the oldest observations are removed from the system while the latest observations are being recorded. Let 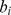 be an indicator of primary user arrival on channel 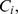 where 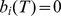 indicate no arrival and 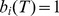 indicate the arrival of the primary users on channel 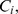 respectively at observation time instance 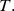 The arrival rate of the primary user 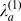 on available channel set 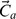 can be estimated as
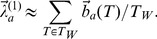
(6)


Based on the information of the other cognitive sensor nodes obtained via macrocell base stations, denoted as 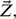 as well as the weighted reliability factors 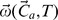 at time instance 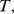 clustering estimation may be conducted at the individual cognitive sensor nodes.

Let 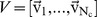 denote the unknown cluster centers in a given network area, and let 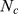 be the number of cluster centers. A cluster center is not a clusterhead but a logical center of the cluster, containing the information about multiple parameters such as common channel availability and the reference interference characteristics of a certain coverage location, all of which indicate similar network behavior such that cognitive sensor nodes can be clustered together. Based on the assumption that a cognitive sensor node can coordinate at least 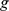 numbers of neighboring cognitive sensor nodes, the maximum number of 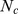 can be initially estimated as 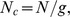 where 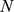 is the total number of cognitive sensor nodes in the given network area. Moreover, let 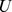 denotes a 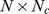 matrix, whose elements 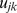 value in 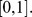 If 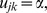 which indicates the membership, i.e., the probability, of the cognitive sensor node 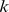 in cluster 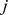 is 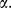 An individual cognitive sensor node obtains the estimates of both the cluster centers and the membership 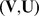 by minimizing the weighted FCM objective function:
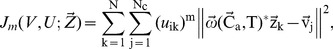
(7)

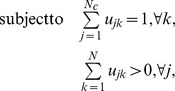
(8)where 
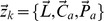
 pertaining to the cognitive sensor node 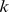 contains location 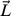, channel availability 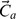 and the corresponding interference characteristics 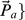, 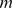 is any real number greater than 1, known as *fuzzifier*, 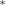 is the transpose, and 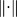 is the Euclidean norm. The estimated cluster centers and the membership of the clusters 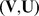 are obtained through loops of estimates for the 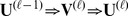 and then checks the termination criterion 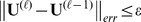, where 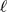 denotes the loop. Accordingly, each element 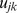 and 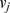 are updated iteratively based on the following Lagrangian multipliers [25]:



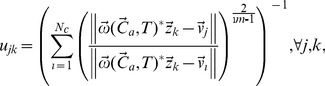
(9)

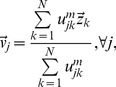
(10)


until 
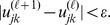


The steps for the proposed cluster estimation are summarized in Table 1.

**Table 1:** Clustering Estimation.

1: (**ESTIMATION**)
2: Initialize *l* = 0, and **U** ^(*l*)^ = [**u** *_jk_*];
3: **if** need clustering **then**
4: Retrieve cognitive sensor node information Z;
5: Retrieve the updated *λ_a_* ^(1)^ using Eq. (6);
6: Update the reliability of the information with Eq. (5) as ω(*C_a_*,*T*)^*^ *Z_k_*;
7: Update the interference characteristics by using Eq. (4) with updated *λ_a_* ^(1)^;
8:** repeat**
9: Update *l* = *l+*1;
10: Calculate the cluster center **V** ^(*l*)^ based on **U** ^(*l-*1)^ using Eq. (9);
11: Update **U** ^(*l*)^ = [**u** *_jk_*] based on **V** ^(*l*)^ using Eq. (10);
12:**until** ||**U** ^(*l*)^−**U** ^(*l-*1)^||*_err_≤ε*;
13: Obtain **U**←**U** ^(*l*)^;
14: **end if**

### 4.3 Cluster formation

At the beginning of the cluster formation stage, the obtained matrix 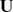 provides information regarding the possible formation of the clusters. To avoid having all cognitive sensor nodes within a cluster competing to be a clusterhead, each cognitive sensor node calculates the value of 
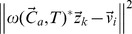
, which indicates the distance to the logical cluster center. The cognitive sensor nodes with the lowest value effectively acts as the clusterhead. It is reasonable since this cognitive sensor node is close to the logical cluster center. In the case where there are several cognitive sensor nodes identifying themselves with the lowest distance values, these selected candidate cognitive sensor nodes compete to be the clusterhead that provides service for the cluster. The most common way to deal with the clusterhead competition is to choose the one with the highest identification (ID) [26]. The clusterhead competition procedure at each cognitive sensor node proceeds according to Table 2.

**Table 2:** Cluster Formation.

**1: State** (**FORMATION**)
2: Listen on the available channels to gain knowledge of competing cognitive sensor nodes for cluster;
3: **if** hear no on-going transmission & never sent HEADCOMPETE message **then**
4: Broadcast HEADCOMPETE.[ ν*_j_*,ID] message with the cluster ID set as the corresponding value of ν*_j_* and cognitive sensor node unique ID;
5:**else**
6:** if** hear on-going transmission = = HEADCOMPETE message **then**
7:** if** own cognitive sensor node ID < HEADCOMPETE.ID **then**
8: Stay silence;
9:** else**
10: Broadcast HEADCOMPETE.[ ν*_j_*,ID] message with its own ID;
11:** end if**
12:**end if**
13:**else**
14:** if** hear no on-going transmission & sent HEADCOMPETE message **then**
15: Clusterhead competition succeed;
16: Broadcast HEAD.[ ν*_j_*,ID] message with unique cluster ID, and its own ID;
17:** end if**
18:**else**
19:** if** hear returning primary users **then**
20: Set to SWITCH state;
21:** end if**
22: **end if**

The most important aspect of the clusterhead competition process is to have at least one cognitive sensor node being declared as the clusterhead. The winning clusterheads broadcast their own IDs as the unique cluster IDs, while the losing cognitive sensor nodes as well as other cognitive sensor nodes keeps silent and set their cluster IDs with the corresponding clusters.

### 4.4 Cluster joining

After successful clusterhead competition, the individual cognitive sensor nodes must now join its corresponding clusters within the network. Based on the obtained matrix 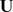, the joining cognitive sensor node 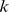 proceeds as follows:

- **Step 1:** Pick index 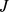 as 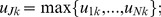

- **Step 2:** Look up the cluster center 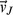 containing the information of available channels;
- **Step 3:** Find the common available channels of 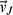 and its own spectrum sensing results;
- **Step 4:** Listen to these channels in order to obtain HEAD.[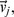
ID] message or control messages that contains the cluster ID and cluster head ID; [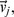
ID];
- **Step 5:** Send a JOIN message on one of the common available channels after identifying no ongoing transmission in the cluster to avoid interference to the cluster members;
- **Step 6:** The joining process succeeds at the 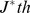 highest value of membership, 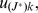 after receiving an acknowledgement message from the cluster head; otherwise, choose the next cluster candidate based on the descending value of 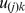 and go back to Step 2.

In the worse-case scenario where a cognitive sensor node fails to join any cluster, the joining cognitive sensor node initializes its own cluster based on the cluster formation process to claim itself as the cluster head.

### 4.5 Cluster switching

Thanks to the matrix 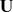, the cognitive sensor node is able to have information of the cluster membership, i.e., the probabilities of belonging to the cluster, which not only facilitates for joining clusters in the highly dynamic network environment but also provides fast backup resolution of returning primary users. As long as returning primary users are identified on the channels that are currently used by a cluster, the cognitive sensor nodes belonging to this cluster immediately switches to the other clusters according to the descending order of 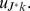 At a same time, a new matrix 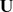 will be calculated to prepare for the next set of joining attempts. This fast switching process is designed to make time for the cognitive sensor node to update 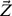 and calculate the new matrix 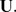 If the switching process succeeds before the new results of the membership information, this new matrix 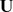 can be used as a reference of the next fast switching process. The switching process is summarized in Table 3.

**Table 3:** Switch.

**1: State** (**SWITCH**)
2: Listen on the channels of the current cluster;
3: **if** returning primary users are identified **then**
4: Call the function of **Clustering** in Algorithm 1;
5:** while** new clustering results (**V,U**) is not ready **do**
6: Fast switch to the other cluster start from (*J* ^*^+1)^th^ value of descending order of *u*_(*j*)*k*_;
7: Perform **Joining** process from Step 2 to 6;
8:** if** all joining attempts fail **then**
9: Stay idle and wait for the new matrix **U**;
10:** else**
11: Finish switch;
12: Break;
13:** end if**
14:** end while**
15: Perform the complete **Joining** process;
16: **end if**

## Results and Discussion

In this section, we present a series of simulation results to evaluate the performance of the proposed multi-parametric clustering scheme. For comparison purposes, a number of previously reported clustering schemes were also evaluated, such as a voting-based clustering algorithm [13] and Spectrum Opportunity-Based Clustering scheme [14]. In line with a large disaster response scenario, we simulate a 500 m×500 m network area uniformly distributed with the cognitive sensor nodes, which forms an ad hoc network. Each cognitive sensor node has a radio transmission range radius of 100 m forming a non fully-connected topology, where not all cognitive sensor nodes are within the transmission range of each other. For every transmission, the one-way propagation delay is set to 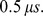 The primary user network dynamics is simulated as a M/G/1 model on total 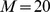 channels with primary user packet arrival rate 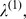 and the channel availability varies accordingly. Each simulation consists of 10 trials, and the results represent the mean of the trials within a 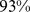 confidence interval.

In the first set of simulations, we study the clustering performance of the proposed scheme. In the second and third set of simulations, we look into the performance of the proposed scheme in the state of cluster formation by comparing with other schemes, as well as the performance of cluster joining, respectively. In the fourth set of simulations, the interference amongst the cluster heads is investigated to give an insightful view of the effectiveness of the proposed scheme. In the last set of simulations, we study the effects on the dynamics of primary users network. The performance measurements are defined as follows:

- Cluster head selection overhead 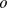: average time consumed on cluster head selection.
- Cluster joining estimation error : ratio of the number of failed cluster joining attempts to the number of attempts.
- Interference probability of clusterheads 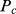: probability of interference between clusterheads.
- Probability of reclustering 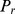: ratio of the reclustering process to the number of instances of network behavior change.

### 5.1 Snapshot of clustering

**Figure 2:**
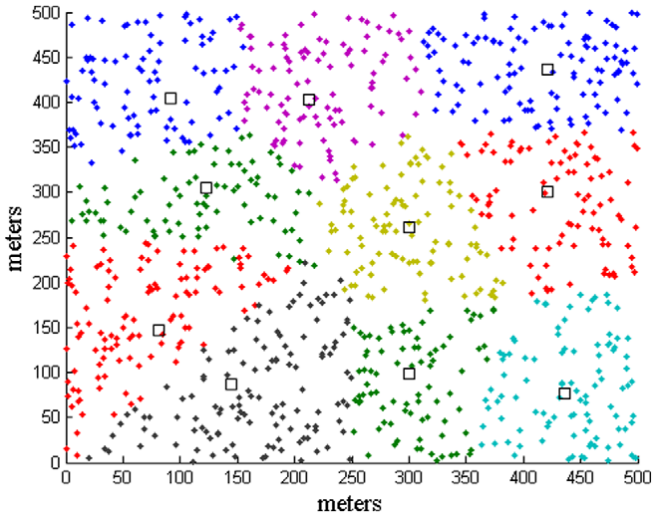
A snapshot of clustering using the proposed scheme for a large scale deployment of cognitive sensor nodes, with different colored dots representing individual cognitive sensor nodes in different clusters, and the small black squares representing the logical centers of the clusters.

A snapshot of clustering obtained using the proposed scheme for a large scale deployment of cognitive sensor nodes is shown in Fig. 2, where there are 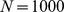 cognitive sensor nodes and the number of clusters is set to a small number 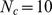 in this set of simulations for the purpose of clarity in the diagram presentation. The cognitive sensor nodes are colored with different colors to indicate that they belong in different clusters, and the corresponding logical centers of the clusters are plotted as small black squares to distinguish them from the cognitive sensor nodes. It can be seen that the boundaries of each cluster are never regular and could be overlapped. It is due to the fact that both of channel availability and interference characteristics are adaptively taken into account in the clustering process. Moreover, the logical cluster centers represent the centers of all the information, denoted as 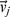 in Eq. 10, and they are not necessarily the physical clusterheads. The performance of the selection of clusterhead is presented in the next section.

### 5.2 Evaluation of cluster formation

**Figure 3:**
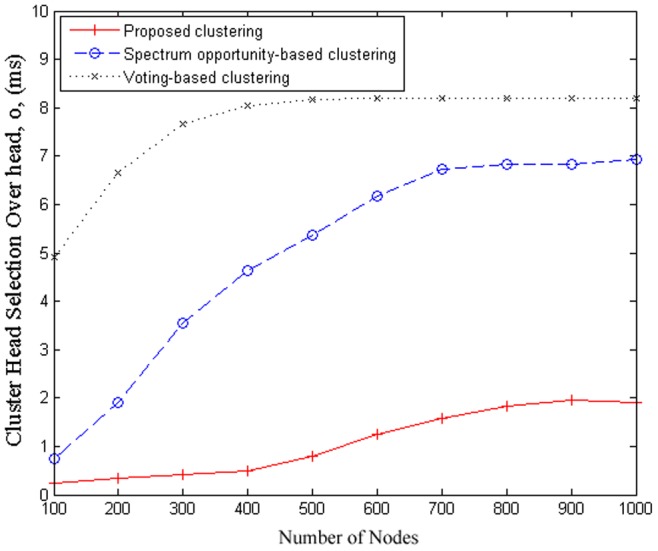
Cluster head selection overhead vs. number of cognitive sensor nodes in comparison.

In this set of simulations, we compare the performance of the proposed clustering scheme with the other schemes under consideration in the study using the cluster head selection overhead, 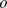, which is defined as the average time consumed on cluster head selection for a cluster in the network in the cluster formation process. Fig. 3 shows that the average clusterhead selection overhead with respect to the number of cognitive sensor nodes. It can be seen that the cluster head selection overhead increases as the number of cognitive sensor nodes increase. Furthermore, it can be observed that the proposed clustering scheme achieves lower overhead than that of the spectrum opportunity-based clustering and voting-based clustering schemes. This is due to the fact that the proposed scheme only considers cognitive sensor nodes within close distance of the estimated logical cluster centers as potential candidates for clusterheads, and as such allow for fewer cognitive sensor nodes to enter into clusterhead competition. This in turn mitigates a massive communication overhead in the clusterhead selection process. This low overhead is very essential for successful integration into large scale cognitive sensor node deployments in cognitive wireless sensor networks.

**Figure 4:**
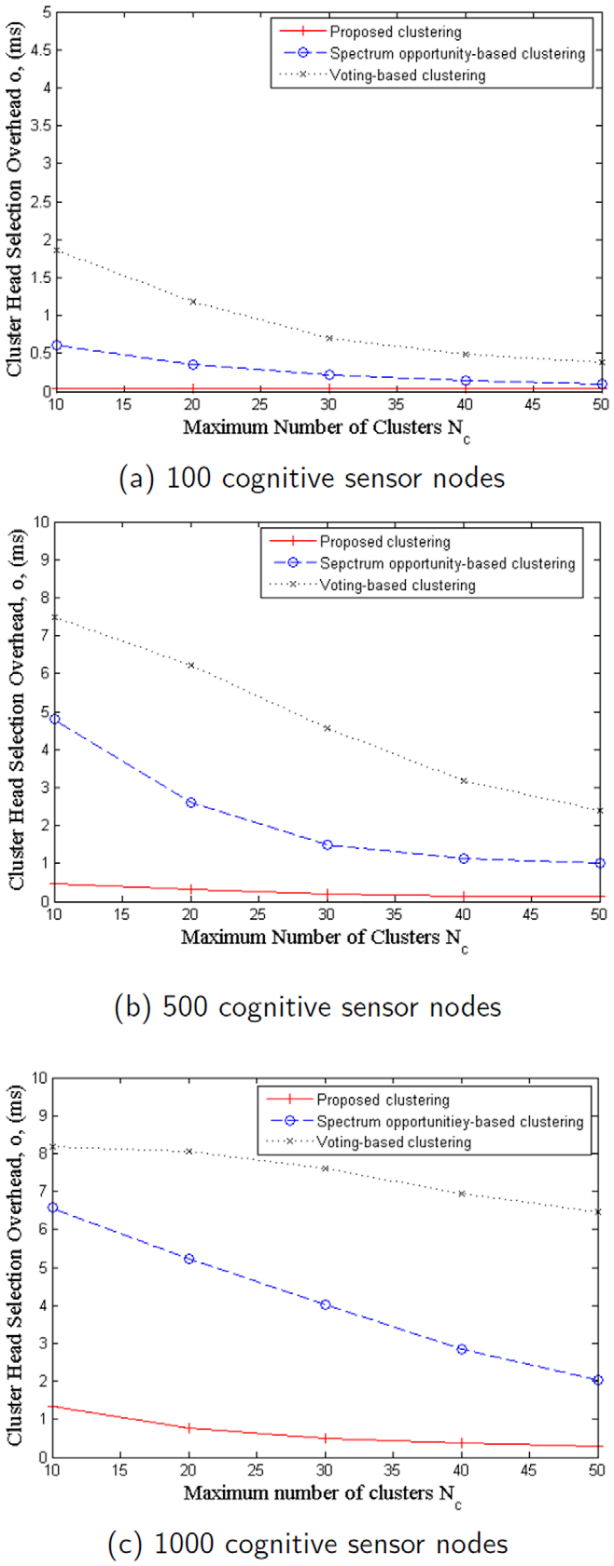
Cluster head selection overhead vs. maximum number of clusters in comparison. (a) 100 cognitive sensor nodes, (b) 500 cognitive sensor nodes, and (c) 1000 cognitive sensor nodes.

To provide a better indication of efficiency, we compare the clusterhead selection overhead 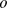 with different number of clusters and fixed number of cognitive sensor nodes. Note that all the three clustering algorithms dynamically determine the number of clusters and as such the actual number of clusters are difficult to tracked and compared in a parametric analysis manner. Therefore, for comparison purposes, the maximum number of clusters is set to the same for each clustering algorithm. Fig. 4 show the simulation results on 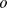 with respect to the maximum number of clusters 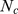 with number of cognitive sensor nodes set to 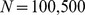, and 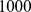, respectively. It can be seen that the amount of clusterhead selection overhead decreases as the number of maximum number of cluster increases; moreover, the proposed scheme has noticeably lower overhead and the results decrease more slowly. It is due to the fact that less cognitive sensor nodes join cluster head competition after estimating their memberships using the proposed scheme.

### 5.3 Evaluations of joining process

**Figure 5:**
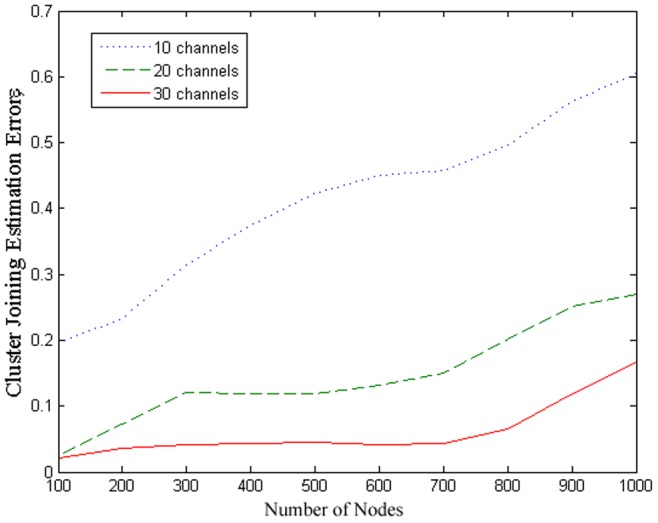
Cluster joining estimation error with different number of channel and different number of cognitive sensor nodes.

To investigate the efficiency of the joining process of the proposed clustering scheme, we study the cluster joining estimation error, which is defined as the ratio of the number of failed cluster joining attempts to the number of attempts. The simulation results are shown in Fig. 5 with total 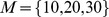 channels versus the different number of cognitive sensor nodes. It can be seen that with the increase of the number of cognitive sensor nodes, the cluster joining estimation error increase. It is due to the large number of cognitive sensor nodes introducing noise in the clustering estimation process, which may lead to estimation discrepancy. Moreover, the number of total channels has noticeable impact on the performance, since the cluster joining estimation error increases when the total number of channels in the network decreases. It is due to the fact that given the same volume of primary traffic within the network, the fewer the channels the higher the usage on each channels. This results in low probability of channel availability and high probability of returning primary users, which effectively increases the interference characteristics and thus increasing the estimation error in the joining process.

### 5.4 Interference

**Figure 6:**
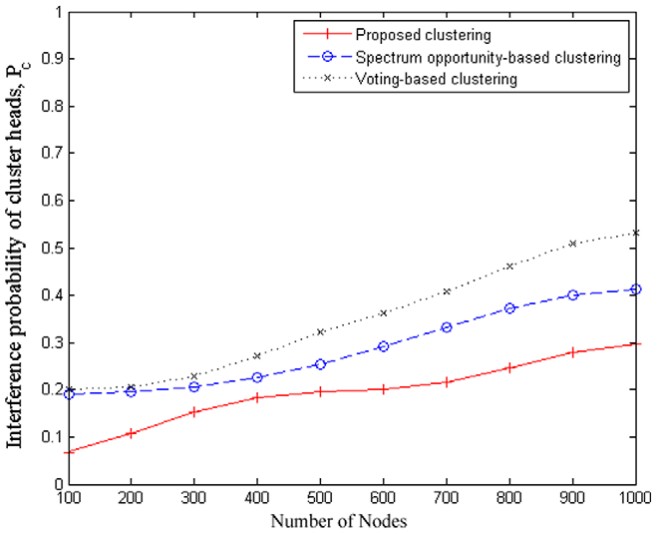
Interference probability of cluster heads with different number of cognitive sensor nodes in comparison.

In this set of simulations, we further compare the performance of the proposed scheme with other clustering algorithms under consideration in the study using interference probability of cluster heads 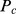, which is defined as the probability of cluster heads interference with each other in the network. Fig. 6 plots the interference probability of cluster heads versus the different number of cognitive sensor nodes in the network area. It can be observed that the interference probability of clusterheads of all three approaches increases as the number of cognitive sensor nodes increases. It can also be observed that the proposed clustering scheme outperforms the other clustering algorithms in the scenario of large scale cognitive sensor node deployments. It is due to two factors: 1) the proposed scheme takes interference characteristics into account as part of the set of network parameters upon which clustering is determined, and 2) unlike the other clustering algorithms, which require each cluster member to be within the transmission range of each other, the proposed clustering scheme only requires the clusterhead to be able to communicate with the cluster members.

### 5.5 Impact from primary dynamics

**Figure 7:**
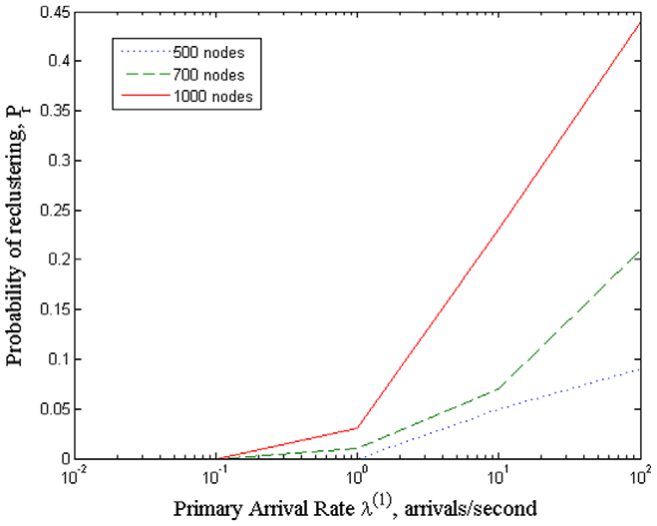
Probability of reclustering with different number of cognitive sensor nodes versus the primary arrival rate 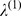.

Finally, the impact from primary dynamics is investigated in this set of simulations by evaluating the probability of reclustering 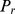, which is defined as the ratio of the number of reclustering to the number of instances of network behavior changes which are caused by the primary user arrivals. Fig. 7 shows the probability of reclustering with different number of cognitive sensor nodes versus the primary arrival rate 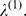 It can be observed that as the primary arrivals increase the probability of reclustering increases. It is clearly due to the fact that the primary user arrivals cause changes to the channel usage status as well as the interference characteristics in the network. As the primary user arrivals increase, the changes of network status occur more frequently, which resulting in the need for more reclustering. Moreover, it also can be seen that the larger the number of cognitive sensor nodes, the higher the probability of reclustering.

### 5.6 Relationship to energy conservation and consumption

One of the objectives of employing clustering strategies for aiding in the coordination of cognitive wireless sensor networks is to improve energy conservation by reducing the amount of communication needed by limiting communications to the clusterheads and the central source. Since much of the energy in WSNs is spent during communication, the clustering process itself should be kept to a minimum to minimize energy costs. As such, there is a direct relationship between energy consumption and clustering overhead (the higher the clustering overhead, the more energy is consumed by the sensor node). Furthermore, the amount of energy consumed by sensor nodes is also directly related to the probability of interference, since a higher probability of interference results in a higher packet losses and hence additional energy to be spent at the sensor nodes for resending packets. It can be seen from Fig. 3 that the proposed scheme achieves lower overhead than that of the spectrum opportunity-based clustering and voting-based clustering schemes, which equates to reduced communication and hence reduced energy consumption. Furthermore, it can be seen from Fig. 6 that the proposed scheme results in the lowest probability of interference compared to the spectrum opportunity-based clustering and voting-based clustering schemes, which also results in reduced energy consumption by requiring less packets to be resend due to interference.

### Conclusions

In this paper, a novel multi-parametric clustering scheme designed for cognitive wireless sensor networks is introduced. Extensive performance evaluation studying the impact on clustering overhead, cluster joining estimation error, interference probability, as well as probability of reclustering, demonstrated that the proposed clustering scheme has strong potential for improving performance of cognitive wireless sensor network deployments with high dynamics and heterogeneity. One point of interest for further investigation is the challenges associated with real-world implementation and deployment of the proposed scheme in cognitive wireless sensor networks. As modern healthcare monitoring systems have increasingly powerful low-power embedded microprocessors for processing sensing data, it is of great interest to implement the proposed scheme to work directly with such existing microprocessors.
